# The Association Between Per- and Polyfluoroalkyl Substances Exposure and Thyroid Hormones in Men and Non-Pregnant Women: A Systematic Review and Meta-Analysis

**DOI:** 10.3390/toxics13030214

**Published:** 2025-03-14

**Authors:** Bin Zhang, Meizi Zhao, Xiangru Cong, Chunyu Liu, Chaofei Li, Yu Qiu, Sha Li, Yanying Chen, Xiaoxue Li, Penghui Li

**Affiliations:** 1 School of Environmental Science and Safety Engineering, Tianjin University of Technology, Tianjin 300384, China; 15588245258@163.com (B.Z.); congxiangru719@163.com (X.C.); liuchunyu981002@163.com (C.L.); 2 Tianjin Eco-Environmental Monitoring Center, Tianjin 300191, China; zhaomz_2004@126.com; 3 Zhonghuan (Fujian) Environmental Technology Co., Ltd., Fuzhou 350025, China; lichaofei1985@hotmail.com (C.L.); fzhkjc@163.com (S.L.); chenyanying_60@126.com (Y.C.); 4 Fujian Jinhuang Environmental Protection Technology Co., Ltd., Fuzhou 350025, China; qiuyu075@163.com; 5 Disaster Medicine Research Center, Medical Innovation Research Division of the Chinese PLA General Hospital, Beijing 100853, China

**Keywords:** per- and polyfluoroalkyl substances, thyroid hormones, non-pregnant women, meta-analysis

## Abstract

Studies have shown that per- and polyfluoroalkyl substances (PFAS) may impact thyroid function in human health. While the consistency between PFAS exposure and thyroid health effects in pregnant women has been validated, the effects on men and non-pregnant women remains inconclusive. To address this, a meta-analysis was carried out in this paper, with 14 eligible studies retrieved from Embase, PubMed, and Web of Science that were published up to 2 June 2024, focusing on the relationship between PFAS exposure and its effect on thyroid hormone levels in the human body. The thyroid function indexes analyzed included thyroid stimulating hormone (TSH), triiodothyronine (T3), thyroxine (T4), free T3 (FT3), and free T4 (FT4). The estimated value (β) and the corresponding confidence interval (95% CI) were extracted from the literature. A heterogeneity test was carried out, and the sensitivity analysis and publication bias of the studies were analyzed using Stata 18.0. The results revealed that in men and non-pregnant women, PFOA was positively correlated with FT3 (β = 0.011, 95% CI = 0.001, 0.02, I^2^ = 13.4). However, no significant associations were found between exposure to other PFAS and thyroid hormones. A subgroup analysis further indicated that the correlations between PFAS exposure and thyroid hormone levels were more significant in adolescents, in both America and Europe.

## 1. Introduction

The normal synthesis and release of thyroid hormone are crucial for human growth and development. Research shows that the impaired signaling of thyroid hormone (TH) and thyroid hormone receptor beta (TRβ) in the liver can trigger hepatic steatosis [1]. Thyroid hormones are of great significance for the development of the brain (including the cerebellum) [2]. A deficiency of thyroid hormones can lead to abnormal cerebellar development, and the abnormalities caused by perinatal hypothyroidism are difficult to fully recover from [3]. At the same time, thyroid hormones and insulin jointly regulate glucose metabolism. A decrease in thyroid hormone sensitivity is associated with a decline in body mass index (BMI), total cholesterol (TC), high-density lipoprotein (HDL), and low-density lipoprotein (LDL) [4,5]. The growing evidence in humans and experimental animals shows that the perturbations of thyroid hormone are associated with specific environmental chemical exposure to heavy metals [6], polybrominated diphenylethers (PBDEs), hydroxylated metabolites (OH-PBDEs) [7], per- and polyfluoroalkyl substances (PFAS) [8], and polychlorinated biphenyls (PCBs) [9].

PFAS are persistent organic pollutants that widely exist in the environment. Due to their potential harm to the environment and health, they have become a research focus in public health [10]. This class of compounds has excellent hydrophobicity and oil-repellent properties [11]; since the 1950s, these compounds have been used in a wide range of industrial and consumer products, such as surfactants, fire protection, textiles, food packaging, lubricants, cosmetics, cleaning products, and many other types [12]. Meanwhile, due to their hydrophobic, oil-repellent properties, and other characteristics, PFAS have a long environmental retention time and are not easily decomposed, leading to more significant environmental damage [13,14]. PFAS that are widely used in the environment include perfluorooctanoate (PFOA), perfluorooctane sulfonate (PFOS), perfluorohexane sulfonate (PFHxS), perfluorononanoic acid (PFNA), perfluorodecanoic acid (PFDA), perfluorododecane sulfonate (PFDoDS), and perfluorodecane sulfonic acid (PFDS). PFAS with long chains, such as PFOA, PFNA, PFDA, PFOS, and PFHxS, were more toxic when compared to ultrashort-chain PFAS (TFA) [15]. Research has revealed that the half-life of PFAS in humans varies depending on the length of the carbon chain, and the half-lives of PFOA, PFNA, PFDA, PFOS, and PFHxS are 3–12 years [16,17].

PFAS have been widely detected in various environmental media, locally and globally, such as aquatic systems, terrestrial sediments, and ecological communities [18]. Extensive human exposure to PFAS has also been confirmed by testing of human blood in multiple countries [19,20,21]. Some studies have pointed out that PFAS will continue to bioaccumulate with the amplification of trophic levels in the food chain. Compared with aquatic food chains, the bioaccumulation process of terrestrial food chains is more complex and more harmful. This bioaccumulation enables PFAS to accumulate to biologically relevant concentrations in the human body, potentially activate the proliferator-activated receptor-alpha (PPARα) signaling pathway, trigger oxidative stress, and induce endocrine disruption [22,23,24]. Notably, PFAS exhibit a pronounced interference with thyroid hormone, which are essential for the growth and development of the body [25,26,27].

A meta-analysis of maternal exposures to PFAS on thyroid hormones showed a positive association between thyroid stimulating hormone (TSH) and PFAS [28]. Pregnant women and their fetuses are susceptible to external factors owing to the special physiological changes during pregnancy [29,30]. This might be due to a higher probability of pregnant women ingesting certain foods (such as seafood and meat, which are rich in specific nutrients) that contain elevated levels of PFAS [31]. For men and non-pregnant women, the association between PFAS and thyroid hormone is inconsistent and controversial. A previous study indicated a negative relation between PFAS exposure and triiodothyronine (T3) and thyroxine (T4) in adults [32]. The exposure of adolescents to PFAS was negatively associated with free T4 (FT4) levels [33]. Other epidemiological studies found a positive correlation between PFAS exposure and T4 [34,35], while another study indicated that the lack of conclusive evidence substantiated a specific association between thyroid hormone levels and PFAS [36]. As a result, a meta-analysis of the potential association between PFAS exposure and thyroid hormone levels in men and non-pregnant women (removing pregnant women from the study population) needs to be conducted. Thus, we systematically retrieved relevant studies and performed a quantitative analysis using a meta-analysis model to assess the relationship between PFAS exposure and changes in thyroid levels in men and non-pregnant women. This study carefully screened high-quality research to ensure that the data were reliable and scientific. Rigorous statistical methods were used to accurately reveal the potential association pattern between them.

## 2. Methods

The study was conducted using the methods proposed in the Preferred Reporting Items for Systematic Review and Meta-analysis Protocol (PRISMA-P) statement [37]. It has been registered with PROSPERO (ID 1001533).

### 2.1. Keywords Search

Perfluoroalkyl acids (PFAA) are the most widely studied category of PFAS. In the scientific literature, PFAA is generally divided into two categories: one of these is long-chain compounds containing seven or more carbon–fluorine bonds; the other category is short-chain compounds with fewer than seven carbon–fluorine bonds [38,39]. Meanwhile, long-chain perfluoroalkyl carboxylic acids (PFCAs, C_n_F_2n+1_COOH, *n* ≥ 7) and long-chain perfluoroalkyl sulfonic acids (PFSA_S_, C_n_F_2n+1_SO_3_H, *n* ≥ 6) are the focus of international due to their environmental persistence and bioaccumulation [40,41]. Thus, we chose PFOA, PFNA, PFDA, PFOS, and PFHxS, which belong to the long-chain PFAA, to explore the correlation between PFAS with thyroid hormone (thyroid stimulating hormone (TSH), triiodothyronine (T3), thyroxine (T4), free T3 (FT3), and free T4 (FT4)).

All relevant articles were retrieved from the Embase, PubMed, and Web of Science databases by setting specific keywords and the latest publication date as 2 June 2024. The following keywords were included:

1. PFAS exposure: “fluorocarbons” or “perfluorinated” or “polyfluorinated” or “polyfluoroalkyl” or “perfluoroalkyl” or “perfluorochemicals” or “PFAs” or “per-and polyfluoroalkyl substances” or “Perfluorohexane sulfonic acid” or “PFHxS” or “perfluorobutane sulfonic acid” or “Perfluorononanoic acid” or “PFNA” or “perfluorooctanoic acid” or “PFOA” or “perfluorooctane sulfonate acid” or “PFOs” or “Perfluorodecaoic acid” or “PFDA”.
2. Five thyroid hormones: “thyroid hormone levels” or “T3” or “T4” or “TSH” or “thyroid dysfunction” or “hypothyroidism” or “hyperthyroidism” or “FT3” or “FT4”.
3. The keywords should contain “1” and “2”.

To minimize the risk of information omission, all relevant references to this topic were meticulously reviewed by two authors (B.Z. and M.Z.) individually. In the event of discrepancies between their assessments, a third party (P.L.) with expertise in the field was consulted to assist in making the final selection decision. Subsequently, during the data collation and in-depth analysis phases, a rigorous double-check system was adhered to, ensuring the accuracy of the data and the reliability of the analysis outcomes.

### 2.2. Inclusion and Exclusion Criteria

Inclusion criteria:

- The study was conducted with men and non-pregnant women;
- The study design was a cohort or cross-sectional study;
- The study included an association between PFAS exposure (at least one pollutant) and thyroid health effects (at least one metric);
- The study included basic statistical data such as the estimated value beta and the 95% CI;
- English-language articles were used.

Exclusion criteria:

- Duplicate studies;
- Off-topic studies;
- Studies reporting only PFAS exposure or thyroid hormone levels with no association between the two;
- Literature reviews, conference reports, case reports, letters, in vivo studies, in vitro studies;
- Studies with incomplete or unaccountable data.

### 2.3. Data Extraction and Quality Assessment

According to the established search strategy, we selected articles from the database and imported them into the EndNote 21.0 software, automatically eliminating duplicate entries. Subsequently, the titles and abstracts were filtered according to the predefined inclusion and exclusion criteria. After a comprehensive and detailed reading of the retained articles, relevant data were extracted from the eligible articles to construct a feature table. The extracted data covered the following aspects: author name, publication year, study area, study period duration, study population (including their age range), sample number type of PFAS, effect indicator, and adjustment variable information. We independently assessed the quality of each study using the Joanna Briggs Institute (JBI) criteria. The JBI evaluation criteria have a total of eight items, and each item is evaluated as “yes”, “no”, ”unclear”, and “not applicable” (Table S1).

### 2.4. Statistical Methods

The data were processed and analyzed using Stata 18 and Endnote X9 software. Relevant data were meticulously extracted from the included literature to construct a database and subsequently analyzed using the meta-analysis module of the Stata plug-in. We used beta (β) values to represent the combined association between human exposure to PFAs and thyroid hormone levels. The specific statistical methods were as follows: (1) The exposure levels of PFAS were evaluated by means of median (interquartile range (IQR)), while the thyroid function was presented in terms of serum thyroid hormone levels. If the original article presented the percentage change (%ch) along with their 95% confidence intervals (CIs) in thyroid hormones associated with a 2-fold increase in the exposure variable, the formula ln [%ch or upper confidence interval (UCI) or lower confidence interval (LCI)) ÷ 100 + 1] ÷ 2 was employed to calculate the β value and its corresponding 95% CIs. If the original article presented the % difference along with their 95% CIs in thyroid hormones associated with an elevated IQR in the PFAS concentration, the formula ln [(% difference or UCI or LCI) ÷ 100 + 1] was used to calculate the β-value and its 95% CI. We uniformly carried out the conversion to β (95% CI) in strict accordance with the above formula prior to the data summarization process [28]. (2) Heterogeneity test (q-test): When no heterogeneity was found in the included literature (I^2^ < 50% and *p* > 0.1), we used the fixed-effects model; otherwise, we used the random-effects model. The forest plots showed the study’s pooled effect sizes. (3) Subgroup analysis: The original literature was analyzed based on the characteristics, stratified according to the possible confounding factors during the research process, and both the effect sizes and 95% CIs for each subgroup were calculated, respectively. (4) Sensitivity analysis: The included articles were removed successively to obtain the new effect values. The stability of the results and the influencing factors were evaluated. (5) Test and correction for publication bias: The funnel plot was used to observe the size of publication bias. The Begg’s funnel plot and Egger’s linear regression test method were combined to undertake testing (Figures S1–S3). In the test, publication bias was considered if the *p* value < 0.05. Publication bias was considered to be absent if the *p*-value ≥ 0.05 [42].

## 3. Results

### 3.1. Selected Study Results

**Figure 1:**
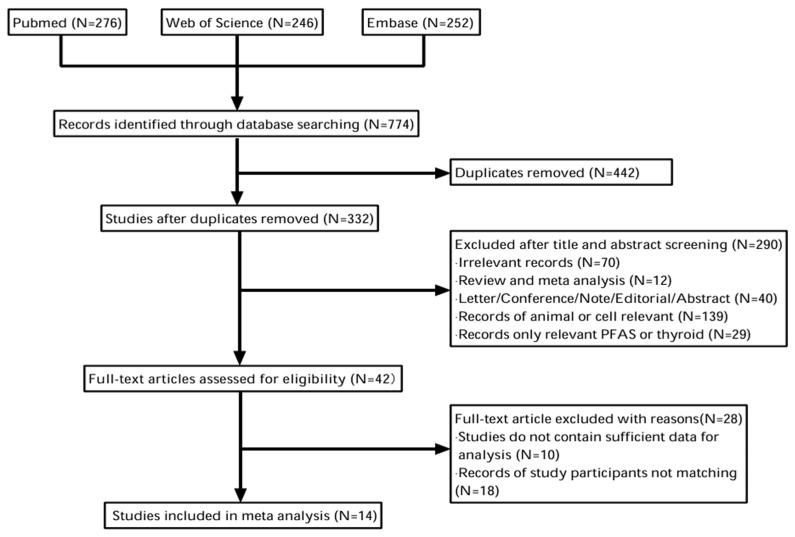
Flowchart of the screening process for meta-analysis.

A total of 774 records were retrieved from three databases (Web of Science—276; Pub Med—246; Embase—252). After 442 duplicate records were removed, 332 study records remained. By screening the titles and abstracts, 234 records were excluded. A total of 46 records were excluded for unrelated population and 42 articles were assessed for full text eligibility, of which 28 were excluded for various reasons. Ultimately, 14 studies were included in the meta-analysis [26,32,33,34,35,43,44,45,46,47,48,49,50,51]. The screening process is shown in Figure 1.

### 3.2. Characteristics of the Study Included

The fourteen cross-sectional studies published between 2010 and 2024 included 38,956 participants [26,32,33,34,35,43,44,45,46,47,48,49,50,51]. Six of the fourteen studies were published in the last five years (2019–2024) [26,32,45,46,47,48]. Four of the fourteen studies were implemented in Europe [26,46,47,49], six in North America [35,43,44,45,50,51], and four in Asia [32,33,34,48]. All the participants of the fourteen studies were over 12 years of age. Additionally, we collected the sample period, exposures, effect indicator, sample detection type (sample detected), and adjustment factors from 14 studies (Table 1 and Table 2).

### 3.3. Association Between PFAs and Thyroid Hormones

**Figure 2:**
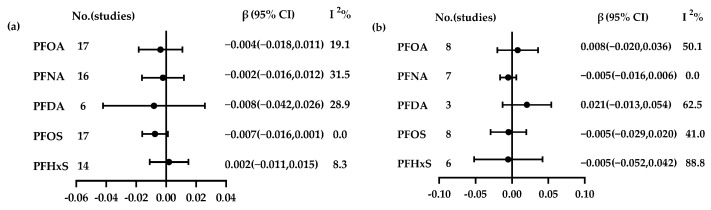

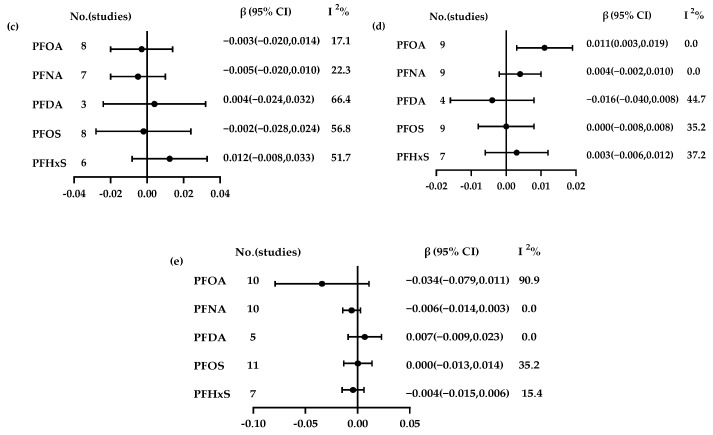
Forest plots showing the associations between PFAS exposure and thyroid hormones: (**a**) PFASs and TSH; (**b**) PFASs and T3; (**c**) PFASs and T4; (**d**) PFASs and FT3; (**e**) PFASs and FT4.

In this meta-analysis, the relationship between exposure to five per- and polyfluoroalkyl substances (PFOA, PFNA, PFDA, PFOS, and PFHxS) and five thyroid hormones (TSH, T3, T4, FT3, and FT4) was analyzed. The number of documents included is shown in Figure 2. The pooled analysis showed a significant effect between PFOA exposure and FT3 (β = 0.011, 95% CI = 0.003, 0.019, I^2^ = 0), but no significant association was found in other PFAS.

### 3.4. Subgroup Analysis

In the comprehensive assessment of the overall effect of PFAS exposure on thyroid hormone levels, potential interference factors were considered. To better understand the effects of PFAS exposure on thyroid hormones under different conditions and to explore potential sources of heterogeneity, a subgroup analysis was carried out with different subgroup sample sizes (>500 and <500), regions (North America, Asia, and Europe), sexes, ages (≥19 and <19), and under the consideration of whether the model was adjusted for BMI.

#### 3.4.1. PFOA

In the subgroup of the sample size > 500, we discovered that PFOA exposure was positively associated with FT3 levels (β = 0.011, 95% CI = 0.001, 0.02, I^2^ = 13.4). However, in the subgroup of the sample size ≤ 500, no significant correlation was found between PFOA exposure and FT3 (β = −0.014, 95% CI = −0.107, 0.079, I^2^ = 0). No significant connection was found between PFOA and other thyroid hormones in the subgroup of the sample size. In the subgroup of North America, PFOA exposure was found to be positively associated with FT3 levels (β = 0.014, 95% CI = 0.005, 0.02, I^2^ = 0), while no association was found in Asia (β = −0.037, 95% CI = −0.124, 0.049, I^2^ = 41.90) and Europe (β = 0.01, 95% CI = −0.012, 0.032, I^2^ = 0). In the subgroup of Europe, we discovered PFOA exposure with a positive effect on T4 levels (β = 0.72, 95% CI = 0.06, 1.38). PFOA exposure was positively associated with FT3 levels (β = 0.014, 95% CI = 0.003, 0.026, I^2^ = 0) in the group without BMI adjustment. In <19 years subgroup, a significant positive association between PFOA exposure and FT3 level (β = 0.014, 95% CI = 0.003, 0.026, I^2^ = 0) was observed, whereas no significant effect was found in the older than 19 years subgroup (β = −0.001, 95 % CI = −0.027, 0.025, I^2^ = 41.5) (Table 3 and Table S2).

#### 3.4.2. PFNA

No significant association was found between PFNA exposure and thyroid hormone levels, which may be due to the small number of relevant studies. A significant positive association between PFNA exposure and T4 levels was observed in the <19 years subgroup (β = 0.278, 95% CI = 0.065, 0.491, I^2^ = 0); no significant effects were found in ≥19 years subgroup (β = −0.005, 95% CI = −0.019, 0.009, I^2^ = 0) (Table 4 and Table S3).

#### 3.4.3. PFDA

In the subgroup of sample size ≤ 500, PFDA exposure was negatively correlated with FT3 levels (β = −0.032, 95% CI = −0.056, −0.007, I^2^ = 0). In the subgroup of the sample size > 500, no significant correlation was found between PFDA and FT3 (β = 0.023, 95% CI = −0.068, 0.114, I^2^ = 0). In the subgroup of Europe, PFDA exposure was found to be negatively correlated with FT3 levels (β = −0.032, 95% CI = −0.056, −0.007, I^2^ = 0). However, no association was found in Asia (β = 0.023, 95% CI = −0.068, 0.114, I^2^ = 57.5) and the data of North America were unable to be found. In the group without BMI adjustment, PFDA exposure was negatively associated with FT3 levels (β = −0.032, 95% CI = −0.056, −0.007, I^2^ = 0), whereas no significant correlation was observed in the group with BMI adjustment (β = 0.023, 95% CI = −0.068, 0.114, I^2^ = 57.5). In the <19 years subgroup (β = −0.018, 95% CI = −0.093, 0.058, I^2^ = 46.5) and ≥19 years subgroup (β = −0.007, 95% CI = −0.026, 0.011), no significant effects were found (Table 5 and Table S4).

#### 3.4.4. PFOS

No significant association between PFOS exposure and thyroid level was found in subgroup analysis. Still, further studies are needed to determine other potential factors (Table S5).

#### 3.4.5. PFHxS

In the non-adjustment of the BMI group, PFHxS exposure was negatively associated with FT3 levels (β = −0.011, 95% CI = −0.021, −0.001, I^2^ = 0), while no significant association was found in the adjustment of the BMI group (β = −0.001, 95% CI = −0.006, 0.003, I^2^ = 58.2) (Table 6 and Table S6).

### 3.5. Sensitivity Analysis and Publication Bias

A sensitivity analysis was carried out to ensure the reliability of the results. In recalculating effect sizes after removing studies individually, no significant change was found in the result (Figure S1), indicating that the results are relatively robust. Meanwhile, the publication bias of the studies was examined by the Begg’s test (Table 7) and Egger’s test (Figures S2 and S3). The Begg’s test results between PFDA and T3 showed publication bias, and the Egger test results between PFHxS and T3 and FT4 showed publication bias (*p* < 0.05). No publication bias was detected in other studies (*p* > 0.05).

## 4. Discussion

This study aims to comprehensively evaluate the association between PFAS exposure and thyroid health risks in men and non-pregnant women. Without considering confounding factors, we discovered that the variation of FT3 levels in men and non-pregnant women was positively connected with PFOA. However, the available evidence was insufficient to establish the impacts of PFAS exposure on alterations in other thyroid hormones. Notably, this result is generally consistent with the results of previous meta-analysis on the effects of PFAS exposure on thyroid hormones in pregnant women [28]. The above findings suggest that the effects of PFAS exposure on human thyroid health may be consistent in pregnant women and the general population (men and non-pregnant women). Further analysis showed that when the sample size, region, age, BMI and other factors were considered, more significant associations were found between PFAS exposure and thyroid hormones.

The available data advanced the possibility that BMI influences the association between PFAS exposure and thyroid hormones [36,52]; thus, we conducted a subgroup analysis depending on whether the data in the paper were adjusted for BMI. In the group without BMI adjustment, it was found that PFAs were significantly correlated with FT3, FT4. However, no significant association was observed in the adjustment of BMI group. The relationship between thyroid hormone levels and weight is clearly recognized, with hyperthyroidism accompanied by weight loss and hypothyroidism by weight gain [53,54]. Some studies have confirmed that individuals outside the normal range of BMI have relatively abnormal thyroid hormone levels [34,55]. This indicates BMI was a confounding factor in the relationship between PFAS exposure and thyroid hormone. Moreover, a cell experiment study has shown that PFAS can stimulate the proliferation and differentiation of adipocytes [56]. It is a possibility that PFAS have an impact on body weight [57].

Previous studies have shown that environmental exposure significantly effects thyroid health [58]. Through subgroup analysis, this study found that the study region may be one key factor influencing PFAS exposure on thyroid health. We found that regarding the effects of PFAS exposure on thyroid hormone levels in Europe and North America, no significant effect was found in Asia. Diet is one of the pathways of environmental exposure to PFAS in humans [59]. Eating habits differ in different geographical locations and cultures. The food category with the highest average concentration of PFAS was “meat and meat products” [60]. Global per capita annual consumption of meat from 2021 to 2023 shows that the top three consuming regions are North America, Oceania, and Europe, accounting for 78.5 kg, 51.4 kg, and 55.6 kg, respectively. Residents of North America and Europe may be exposed to higher concentrations of PFAS, increasing the risk of changes affecting thyroid hormones [61].

In addition, our subgroup analysis also considered participants >19 years old and <19 years old in order to explore the effect of PFAS exposure on thyroid hormone levels. We found that exposure to PFOA and PFNA had a positive correlation with thyroid hormone levels in adolescents (<19). One study found that in areas where drinking water is highly contaminated with PFAS, thyroid hormone levels in adolescents (<19) are significantly affected, especially in pre-adolescent children (2–12) [36]. Adolescence is a stage of continuous body and brain developments, and this makes adolescents more sensitive to thyroid-disrupting chemicals than the adult population [62].

We discovered a positive effect between PFOA and FT3 when data were extracted from studies with sample sizes > 500. No significant correlation was found between PFOA exposure and FT3 with the sample sizes < 500, which indicated that sample size might be a reason for the heterogeneity of the results. In addition, we found no significant effect in PFAS on thyroid hormone levels in subgroups stratified based on sex; a potential possibility for this is that some unknown confusing factors were disturbed.

In subgroup analysis, it was shown that PFOA, PFNA, PFDA, and PFHxS have an association with thyroid hormone; no association was found between PFOS and thyroid hormone. The five PFAS were structurally similar, and the presence or absence of association results may be due to differences in exposure dose [63]. A study of Chinese adults showed that the changing levels of thyroid hormone (T3, T4) plateaued when the amount of PFAS exceeded a critical exposure threshold (about 0.5–10 ng/mL) [32]. Of the 14 studies included in this study, PFAS concentrations ranged from 0.001 to 1 μg/L in five studies [26,34,46,48,49], from 0.1 to 10 μg/L in three studies [44,45,47], and from 1 to 100 μg/L in six studies [32,33,35,43,50,51]. This suggests that differences in PFAS concentrations across the included studies may have contributed to the partial associations observed in the study results. In addition, in two recent studies exploring the relationship between PFAS and health outcomes, a non-monotonic dose–response relationship was found between PFOA (a common PFAS) and diabetes risk. This indicated that the association of exposure dose is not a simple monotonic relationship but a more complex pattern of change [64,65]. In the subgroup analysis of this study, we found a positive correlation between PFAS (PFOA, PFNA) and thyroid hormone, and a negative correlation between PFAS (PFDA, PFHxS) and thyroid hormone. The relationship between PFAS and thyroid hormone levels exhibits significant complexity and diversity, potentially demonstrating a non-monotonic dose–response relationship. Different PFAS showed varying associations with thyroid hormone levels. Subgroup analysis suggests that PFAS may exert potential toxicological effects on thyroid hormones through multiple mechanisms, necessitating further in-depth research to elucidate their specific mechanisms of action and dose–response relationships.

PFAS has the potential to influence thyroid levels in a variety of ways. Investigations have demonstrated that PFAS can bind with transthyretin (TTR) and thyroxine-binding globulin (TBG). TTR and TBG are two major thyroid hormone distributor proteins in human plasma, playing important roles in stabilizing the TH levels [66,67]. The combination of PFAS and distributor proteins can alter the key points of the thyroid axis, disturbing the thyroid hormone levels [68]. Additionally, it has been confirmed that iodide is an essential constituent of thyroid hormones, and NIS-mediated iodide uptake plays a pivotal role in their biosynthesis [69]. PFAS impairs iodine uptake by thyroid cells through the competitive inhibition of sodium iodide symporter (NIS) [70]. Some studies indicate that PFAS induces oxidative damage, disrupting normal immune function [71,72]. PFAS can increase the generation of reactive oxygen species (ROS) in vivo [73]. The accumulation of oxygen free radicals may inhibit thyroid peroxidase (TPO) activity, thereby interfering with thyroid hormone production and contributing to the development of hypothyroidism [74].

Still, there are limitations to this systematic review and meta-analysis. Firstly, according to our standard screening literature, the number of studies on specific PFAS in thyroid hormone levels is small, which may lead to heterogeneity between studies. Although subgroup analysis was performed, the leading causes of heterogeneity remain unclear. Secondly, the included studies were cross-sectional. When observing the relationship between exposures and outcomes, they were often subject to interference from multiple confounding factors. These factors may be simultaneously associated with exposure and health outcomes, thereby masking or distorting the associations. Therefore, a cautious and rigorous attitude should be adopted towards the conclusions of this study. Finally, we only stratified subgroups based on whether BMI was adjusted in the original literature, without considering other adjustment factors. If other adjustment factors are considered, the number of eligible articles will be small, which may lead to a less obvious significance in the results and some key information being ignored. Further large cohort studies with adequate and reliable exposure data should be conducted to better understand the association between PFAS exposure and thyroid hormone levels. In addition, with sufficient data, multiple comprehensive adjustment factors should be considered.

## 5. Conclusions

This study revealed a stable association between PFOA and FT3 in men and non-pregnant women. Subgroup analysis showed that sample size, region, BMI, and age were confounding factors affecting the association between exposure to PFAS and thyroid hormone changes. In particular, the correlation is more significant in studies with larger sample sizes and in studies from Europe and North America. In addition, it is of great concern that adolescents were more sensitive to PFAS exposure in men and non-pregnant women, which provides an important direction for follow-up research and related health risk assessment.

## Figures and Tables

**Table 1:** Characteristics of studies included in the meta-analysis.

Author (Year)	Region	Period	Sample Size	Age	Design
Carmen Freire (2023)	Spain	2017–2019	129	15–17	cross-section
Elisa Gallo (2022)	Veneto	2017–2019	21,424	14–39	cross-section
Yanan Xing (2024)	China	2017–2018	10,853	≥18	cross-section
Kai Tan (2024)	China	May 2023–June 2023	746	>60	cross-section
Andrea Rodríguez-Carrillo (2023)	Belgium, Slovakia, Spain	2014–2020	733	12–19	cross-section
Élyse Caron-Beaudoin (2019)	Quebec	May 2015–October 2015	186	3–19	cross-section
Srishti Shrestha (2015)	New York	2005	84	55–74	cross-section
Samuel C. Byrne (2018)	Alaska	2013–2014	85	18–45	cross-section
Hélène Tillaut (2022)	France	2014–2018	476	12	cross-section
Michael S. Bloom (2010)	New York	2006	31	31–45	cross-section
Kyunghee Ji (2012)	Korea	July 2008–August 2008	633	12–75	cross-section
Lili Wen (2013)	USA	2007–2010	1181	≥20	cross-section
Glenys M. Webster (2016)	USA	2007–2008	1525	≥18	cross-section
Linna Xie (2024)	China	2018	836	11–15	cross-section

**Table 2:** Exposure, effect indicator, and adjustment factors.

Author (Year)	Sample Detected	Exposures	Effect Indicator	Adjustment Factors
Carmen Freire (2023)	serum	PFOA, PFNA, PFDA, PFOS, PFHxS	T3, FT4, TSH	Child passive smoking, alcohol intake, total fish intake, iodine intake, physician-diagnosed thyroid disease history, maternal schooling, and the use of current or recent medication
Elisa Gallo (2022)	serum	PFOA, PFNA, PFOS, PFHxS	TSH	BMI, time lag between the enrolment and the beginning of the study, gender, physical activity, smoking habits, food consumption, country of birth, alcohol consumption, education level, and laboratory in charge of the TSH analyses
Yanan Xing (2024)	serum	PFOA, PFNA, PFDA, PFOS, PFHxS	T4, T3	Age, BMI, sex, nationality, education level, residence, marital status, annual family income, current smoking, current alcohol consumption, and multivitamin supplementation
Kai Tan (2024)	serum	PFOA, PFNA, PFDA, PFOS, PFHxS	TSH, T3, T4, FT3, FT4	Sex, age, BMI, smoking, and alcohol
Andrea Rodríguez-Carrillo (2023)	serum	PFOA, PFNA, PFOS	TSH, FT3, FT4	Random effect, age, sex, z-BMI, household education, and urinary creatinine concentration
Élyse Caron-Beaudoin (2019)	serum	PFOA, PFNA, PFOS, PFHxS	TSH, FT4	Age, sex, studied nation, urinary iodine, urinary cotinine, parent’s education, and BMI z-score
Srishti Shrestha (2015)	serum	PFOA, PFOS	TSH, T3, T4, FT4	Age, sex, years of education, and serum ∑ PCBs
Samuel C. Byrne (2018)	serum	PFOA, PFNA, PFOS	TSH, T3, T4	Using total T3, fT3, total T4, fT4 or TSH as the dependent variables, adjusted for age, sex, and smoking habits
Hélène Tillaut (2022)	serum	PFOA, PFNA, PFDA, PFOS, PFHxS	TSH, FT3, FT4	Namely parental history of thyroid hormonal disorders, season, and time of day for the blood draw
Michael S. Bloom (2010)	serum	PFOA, PFNA, PFDA, PFOS, PFHxS	TSH, FT4	Age, gender, BMI, cigarette smoking, history of physician-diagnosed goiter or thyroid condition, race/ethnicity, the use of medication, and the consumption situation of sportfish from self-report
Kyunghee Ji (2012)	serum	PFOA, PFNA, PFDA, PFOS, PFHxS	TSH, T4	Age, sex, and BMI
Lili Wen (2013)	serum	PFOA, PFNA, PFOS, PFHxS	TSH, T3, T4, FT3, FT4	Age, gender, race, alcohol consumption, smoking status, and urinary iodine
Glenys M. Webster (2016)	serum	PFOA, PFNA, PFOS, PFHxS	TSH, T3, T4, FT3, FT4	Rage, race, log serum cotinine, sex, parity, pregnancy, and menopause status
Linna Xie (2024)	serum	PFOA, PFNA, PFDA, PFOS, PFHxS	TSH, FT3, FT4	Sex, age, BMI, and household income levels

**Table 3:** Subgroup associations of PFOA exposure with thyroid health effects.

Subgroup		FT3			FT4		
		No	β (95% CI)	I^2^	No	β (95% CI)	I^2^
Sample size	<500	3	−0.014 (−0.107, 0.079)	0.0	4	0.006 (−0.028, 0.049)	0.0
	>500	6	**0.011** (**0.001, 0.020**)	13.4	6	−0.057 (−0.118, 0.005)	94.7
Region	Asia	2	−0.037 (−0.124, 0.049)	41.9	2	−0.760 (−2.250, 0.730)	98.2
	North America	4	**0.014** (**0.005, 0.022**)	0.0	6	−0.001 (−0.017, 0.014)	0.0
	Europe	3	0.010 (−0.012, 0.032)	0.0	2	−0.045 (−0.146, 0.056)	92.1
Adjusted for BMI	yes	2	−0.037 (−0.124, 0.0491)	41.9	3	−0.445 (−0.896, 0.006)	96.4
	no	7	**0.013** (**0.005, 0.021**)	0.0	7	−0.017 (−0.055, 0.020)	85.9
Sex	male	9	0.001 (−0.006, 0.007)	38.0	9	−0.003 (−0.020, 0.014)	84.3
	female	9	0.002 (−0.003, 0.006)	32.8	10	0.000 (−0.007, 0.007)	64.0
Age	<19	5	**0.014** (**0.003, 0.026**)	0.0	4	−0.152 (−0.289, −0.015)	96.2
	≥19	4	−0.001 (−0.027, 0.025)	41.5	7	−0.004 (−0.017, 0.010)	0.0

Bold font: used to highlight associated results.

**Table 4:** Subgroup associations of PFNA exposure with thyroid health effects.

Subgroup		TSH			T3			T4		
		No	β (95% CI)	I^2^	No	β(95% CI)	I^2^	No	β (95% CI)	I^2^
**Age**	<19	7	0.005 (−0.012, 0.021)	0.0	1	0.020 (−0.030, 0.080)	--	2	**0.278** (**0.065, 0.491**)	0.0
	≥19	5	−0.000 (−0.061, 0.060)	53.4	5	−0.005 (−0.017, 0.008)	0.0	5	−0.005 (−0.019, 0.009)	0.0

Bold font: used to highlight associated results; (--): no value.

**Table 5:** Subgroup associations of PFDA exposure with thyroid health effects.

Subgroup		TSH				T3				T4		
		No	β (95% CI)		I^2^	No	β(95% CI)		I^2^	No	β (95% CI)	I^2^
Age	<19	3	−0.018 (−0.093, 0.058)		35.3	1	0.040 (−0.040, 0.120)		--	1	−0.080 (−0.660, 0.500)	--
	≥19	2	**−0.022** (**−0.041, −0.003**)		0.0	1	−0.001 (−0.022, 0.019)		--	1	−0.007 (−0.027, 0.130)	--
Subgroup			**FT3**					**FT4**				
			**No**	**β** **(95%CI)**			**I^2^**	**No**		**β** **(95%CI)**		**I^2^**
Sample size	<500		2	**−0.032** (**−0.056, −0.007**)			0.0	3		0.014 (−0.012, 0.041)		0.0
	>500		2	0.023 (−0.068, 0.114)			57.5	2		0.003 (−0.016, 0.023)		0.0
Region	Asia		2	0.023 (−0.068, 0.114)			57.5	2		0.003 (−0.016, 0.023)		0.0
	North America		0	--			--	1		0.090 (−0.020, 0.210)		--
	Europe		2	**−0.032** (**−0.056, −0.007**)			0.0	2		0.010 (−0.017, 0.037)		0.0
Adjusted for BMI	yes		2	0.023 (−0.068, 0.114)			57.5	3		0.011 (−0.024, 0.046)		7.6
	no		2	**−0.032** (**−0.056, −0.007**)			0.0	2		0.010 (−0.017, 0.037)		0.0

Bold font: used to highlight associated results; (--): no value.

**Table 6:** Subgroup associations of PFHxS exposure with thyroid health effects.

Subgroup		FT3			FT4		
		No	β (95%CI)	I^2^	No	β (95%CI)	I^2^
Adjusted for BMI	yes	4	0.015 (−0.015, 0.044)	67.0	5	−0.001 (−0.014, 0.030)	0.0
	no	5	0.005 (−0.001, 0.011)	0.0	3	**−0.011** (**−0.021, −0.001**)	**0.0**

Bold font: used to highlight associated results.

**Table 7:** Begg’s test for the associations of PFASs exposure and thyroid hormones.

	TSH		T3		T4		FT3		FT4	
	Z-Value	*p*	Z-Value	*p*	Z-Value	*p*	Z-Value	*p*	Z-Value	*p*
PFOA	0.04	0.967	0.62	0.536	1.08	0.279	1.77	0.076	0.81	0.419
PFNA	1.22	0.224	0.24	0.806	0.52	0.602	0.00	1.000	1.62	0.105
PFDA	0.00	1.000	0.00	1.000	0.34	0.734	0.34	0.734	0.73	0.462
PFOS	0.87	0.386	0.12	0.902	0.89	0.371	0.00	1.000	0.86	0.390
PFHxS	0.05	0.956	0.96	0.339	0.75	0.454	0.46	0.649	2.10	0.035

## Data Availability

The original data presented in the study are included in the article/Supplementary Materials; further inquiries can be directed to the corresponding authors.

## Supplementary Materials

The following supporting information can be downloaded at https://www.mdpi.com/article/10.3390/toxics13030214/s1: Figure S1: Sensitivity analysis; Figure S2: Egger’s Test; Figure S3: Funnel plot; Table S1: Quality Assessment; Table S2: Subgroup associations of PFOA exposure with thyroid health effects; Table S3: Subgroup associations of PFNA exposure with thyroid health effects; Table S4: Subgroup associations of PFDA exposure with thyroid health effects; Table S5: Subgroup associations of PFOS exposure with thyroid health effects; Table S6: Subgroup associations of PFHxS exposure with thyroid health effects.
