# Supplementary material for: The Association Between Per- and Polyfluoroalkyl Substances Exposure and Thyroid Hormones in Men and Non-Pregnant Women: A Systematic Review and Meta-Analysis
Source: Toxics. 2025 Mar 14;13(3):214. doi: 10.3390/toxics13030214 (PMC11946724; doi:10.3390/toxics13030214)
Supplement: Supplementary file 1 [file toxics-13-00214-s001.zip › Supplementary File(s)/Figure S1. S2. S3..docx]

Figure S1. Sensitivity analysis

TSH:(a) PFOA exposure and TSH(b) PFNA exposure and TSH(c) PFDA exposure and TSH(d) PFOS exposure and TSH(e) PFHxS exposure and TSH

(a) (b)

(c) (d)

(e)

T3:(a) PFOA exposure and T3(b) PFNA exposure and T3(c) PFDA exposure and T3(d) PFOS exposure and T3(e) PFHxS exposure and T3

(a) (b)

(c) (d)

(e)

T4:(a) PFOA exposure and T4(b) PFNA exposure and T4(c) PFDA exposure and T4(d) PFOS exposure and T4(e) PFHxS exposure and T4

(a) (b)

(c) (d)

(e)

FT3:(a) PFOA exposure and FT3(b) PFNA exposure and FT3(c) PFDA exposure and FT3(d) PFOS exposure and FT3(e) PFHxS exposure and FT3

(a)

 (b)

(c) (d)

(e)

FT4:(a) PFOA exposure and FT4(b) PFNA exposure and FT4(c) PFDA exposure and FT4(d) PFOS exposure and FT4(e) PFHxS exposure and FT4

(a)

 (b)

(c) (d)

(e)

Figure S2. Egger’s Test

| PFAS | TSH | | | | T3 | | | | T4 | | | | FT3 | | | | FT4 | | | |
| --- | --- | --- | --- | --- | --- | --- | --- | --- | --- | --- | --- | --- | --- | --- | --- | --- | --- | --- | --- | --- |
|  | β | LCI | UCI | P | β | LCI | UCI | P | β | LCI | UCI | P | β | LCI | UCI | P | β | LCI | UCI | P |
| PFOA | 0.0848196 | -0.07044793 | 0.8741184 | 0.807 | 47.5958 | -196.7695 | 291.9611 | 0.617 | 0.1015832 | -2.677492 | 2.880658 | 0.932 | 0.0017369 | -0.0953946 | 0.0988684 | 0.958 | 0.6963635 | -0.0269431 | 1.41967 | 0.057 |
| PFNA | 0.0180159 | -0.0992694 | 0.1353013 | 0.720 | -0.1381532 | -0.2983789 | 0.0220725 | 0.071 | 0.255193 | -2.38343 | 2.893816 | 0.814 | -0.0102145 | -0.0451036 | 0.0246746 | 0.420 | 0.009941 | -0.0186632 | 0.0385453 | 0.389 |
| PFDA | -0.0821344 | -0.2481957 | 0.0839269 | 0.167 | -0.0069706 | -0.0270285 | 0.0130874 | 0.142 | -0.1262944 | -2.925022 | 2.672434 | 0.864 | -0.0172709 | -- | -- | -- | 0.0192395 | -0.4572427 | 0.4957217 | 0.698 |
| PFOS | -0.0198788 | -0.2557703 | 0.2160128 | 0.848 | 22.65173 | -146.4507 | 191.7541 | 0.729 | 0.1208436 | -2.81567 | 3.057357 | 0.923 | -0.0286211 | -0.0777871 | 0.020545 | 0.161 | -0.1433782 | -0.3219989 | 0.0352425 | 0.094 |
| PFHxS | -0.0018537 | -0.0894621 | 0.0857548 | 0.956 | -0.0090386 | -0.0156226 | -0.0024545 | 0.027 | 0.3655359 | -1.049584 | 1.780655 | 0.513 | -0.0388575 | -0.1986973 | 0.1209823 | 0.199 | -0.0941768 | -0.1795888 | -0.0087648 | 0.039 |

Figure S3. Funnel plot

PFOA-TSH

PFNA-TSH

PFDA-TSH

PFOS-TSH

PFHxS-TSH

PFOA-T3

PFNA-T3

PFDA-T3

PFOS-T3

PFHxS-T3

PFOA-T4

PFNA-T4

PFDA-T4

PFOS-T4

PFHxxS-T4

PFOA-FT3

PFNA-FT3

PFDA-FT3

PFOS-FT3

PFHxS-FT3

PFOA-FT4

PFNA-FT4

PFDA-FT4

PFOS-FT4

PFHxS-FT4
