# Supplementary material for: The Association Between Per- and Polyfluoroalkyl Substances Exposure and Thyroid Hormones in Men and Non-Pregnant Women: A Systematic Review and Meta-Analysis
Source: Toxics. 2025 Mar 14;13(3):214. doi: 10.3390/toxics13030214 (PMC11946724; doi:10.3390/toxics13030214)
Supplement: Supplementary file 1 [file toxics-13-00214-s001.zip › Supplementary File(s)/Table S2. S3. S4. S5. S6..docx]

Table S2. Subgroup associations of PFOA exposure with thyroid health effects.

| Subgroup | |  | **TSH** | | | **T3** | | | **T4** | | |
| --- | --- | --- | --- | --- | --- | --- | --- | --- | --- | --- | --- |
|  | |  | **NO** | **β(95%CI)** | **I^2^** | **NO** | **β(95%CI)** | **I^2^** | **NO** | **β(95%CI)** | **I^2^** |
| **Sample size** | | <500 | 7 | 0.032(-0.068,0.132) | 53.2 | 3 | 0.341(-2.906,3.588) | 46.0 | 4 | 0.224(-0.056,0.545) | 39.1 |
|  |  | >500 | 10 | -0.002(-0.012,0.009) | 0.0 | 4 | 0.011(-0.027,0.048） | 48.6 | 5 | -0.002(-0.019,0.015) | 0.0 |
| **Region** | | Asia | 2 | -0.030(-0.061,-0.000) | 0.0 | 1 | -0.001(-0.033,0.031) | -- | 2 | -0.004(-0.036,0.027) | 50.3 |
|  |  | North America | 7 | 0.043(-0.029,0.115) | 47.5 | 5 | 1.207(-1.338,3.752) | 52.4 | 6 | 0.004(-0.023,0.03) | 0.0 |
|  |  | Europe | 8 | 0.001(-0.011,0.014) | 0.0 | 1 | 0.060(-0.030,0.150) | -- | 1 | 0.720(0.060,1.380) | -- |
| **Adjust for BMI** | | yes | 4 | -0.029(-0.06,0.001) | 0.0 | 1 | -0.001(-0.033,0.031) | -- | 3 | -0.002(-0.030,0.027) | 28.9 |
|  | | no | 13 | 0.002(-0.015,0.019) | 26.1 | 6 | 0.04(-0.063,0.143) | 45.2 | 6 | 0.084(-0.076,0.256) | 32.7 |
| **Sex** | | male | 13 | -0.001(-0.01,0.019) | 0.0 | 4 | 0.015(-0.036,0.065) | 20.3 | 4 | 0.050(-0.157,0.256) | 40.9 |
|  | | female | 12 | 0.001(-0.007,0.010) | 0.0 | 3 | 0.174(-0.254,0.601) | 69.1 | 3 | 0.008(-0.038,0.055) | 0.0 |
| **Age** | | <19 | 7 | 0.002(-0.014,0.018) | 0.0 | 1 | 0.060(-0.030,0.150) | -- | 2 | 0.313(-0.303,0.929) | 71.6 |
|  | | ≥19 | 9 | -0.003(-0.031,0.025) | 0.0 | 6 | 0.011(-0.034,0.056) | 47.70 | 6 | 0.006(-0.014,0.027) | 0.0 |
|  | |  |  | | | | | |  | | |
| subgroup | |  | **FT3** | | | | | | **FT4** | | |
|  | |  | **NO** | **β(95%CI)** | | | **I^2^** | | **NO** | **β(95%CI)** | **I^2^** |
| **Sample size** | | <500 | 3 | -0.014(-0.107,0.079) | | | 0.0 | | 4 | 0.006(-0.028,0.049) | 0.0 |
|  |  | >500 | 6 | **0.011(0.001,0.020)** | | | 13.4 | | 6 | -0.057(-0.118,0.005) | 94.7 |
| **Region** | | Asia | 2 | -0.037(-0.124,0.049) | | | 41.9 | | 2 | -0.760(-2.250,0.730) | 98.2 |
|  |  | North America | 4 | **0.014(0.005,0.022)** | | | 0.0 | | 6 | -0.001(-0.017,0.014) | 0.0 |
|  |  | Europe | 3 | 0.010(-0.012,0.032) | | | 0.0 | | 2 | -0.045(-0.146,0.056) | 92.1 |
| **Adjust for BMI** | | yes | 2 | -0.037(-0.124,0.0491) | | | 41.9 | | 3 | -0.445(-0.896,0.006) | 96.4 |
|  | | no | 7 | **0.013(0.005,0.021)** | | | 0.0 | | 7 | -0.017(-0.055,0.020) | 85.9 |
| **Sex** | | male | 9 | 0.001(-0.006,0.007) | | | 38.0 | | 9 | -0.003(-0.020,0.014) | 84.3 |
|  | | female | 9 | 0.002(-0.003,0.006) | | | 32.8 | | 10 | 0.000(-0.007,0.007) | 64.0 |
| **Age** | | <19 | 5 | **0.014(0.003,0.026)** | | | 0.0 | | 4 | -0.152(-0.289,-0.015) | 96.2 |
|  | | ≥19 | 4 | -0.001(-0.027,0.025) | | | 41.5 | | 7 | -0.004(-0.017,0.010) | 0.0 |

**Table S3.** Subgroup associations of PFNA exposure with thyroid health effects.

| Subgroup |  | **TSH** | | | **T3** | | | **T4** | | |
| --- | --- | --- | --- | --- | --- | --- | --- | --- | --- | --- |
|  |  | **NO** | **β(95%CI)** | **I^2^** | **NO** | **β(95%CI)** | **I^2^** | **NO** | **β(95%CI)** | **I^2^** |
| **Sample size** | <500 | 6 | 0.064(-0.004,0.132) | 26.2 | 2 | 0.020(-0.035,0.075) | 0.0 | 2 | 0.102(-0.228,0.432) | 0.0 |
|  | >500 | 10 | -0.006(-0.019,0.006) | 23.9 | 4 | -0.005(-0.017,0.008) | 0.0 | 5 | -0.005(-0.018,0.008) | 0.0 |
| **Region** | Asia | 2 | 0.021(-0.112,0.154) | 71.6 | 1 | -0.015(-0.042,0.012) | -- | 2 | -0.003(-0.023,0.016) | 0.0. |
|  | North America | 8 | 0.029(-0.037,0.094) | 37.4 | 4 | -0.002(-0.016,0.012) | 0.0 | 5 | 0.025(-0.120,0.169) | 56.3 |
|  | Europe | 8 | 0.001(-0.008,0.011) | 0.0 | 1 | 0.020(-0.030,0.080) | -- | 1 | 0.180(-0.210,0.570) | -- |
| **Adjust for BMI** | yes | 8 | -0.008(-0.026,0.009) | 46.2 | 1 | -0.015(-0.042,0.012) | -- | 3 | 0.006(-0.038,0.050) | 67.5 |
|  | no | 9 | 0.014(-0.012,0.039) | 3.2 | 5 | -0.001(-0.014,0.013) | 0.0 | 5 | -0.006(-0.023,0.011) | 0.0 |
| **Sex** | male | 9 | -0.005(-0.019,0.010) | 0.0 | 4 | -0.004(-0.056,0.048) | 43.6 | 5 | -0.023(-0.051,0.005) | 0.0 |
|  | female | 8 | -0.009(-0.030,0.012) | 17.5 | 3 | 1.180(-1.710,4.070) | 56.0 | 3 | 0.017(-0.003,0.038) | 0.0 |
| **Age** | <19 | 7 | 0.005(-0.012,0.021) | 0.0 | 1 | 0.020(-0.030,0.080) | -- | 2 | **0.278(0.065,0.491)** | 0.0 |
|  | ≥19 | 5 | -0.000(-0.061,0.060) | 53.4 | 5 | -0.005(-0.017,0.008) | 0.0 | 5 | -0.005(-0.019,0.009) | 0.0 |
|  |  |  |  |  |  |  |  |  |  |  |
| subgroup |  | **FT3** | | | | **FT4** | | | |  |
|  |  | **NO** | **β(95%CI)** | | **I^2^** | **NO** | **β(95%CI)** | | **I^2^** |  |
| **Sample size** | <500 | 3 | -0.009(-0.035,0.016) | | 0.0 | 4 | 0.008(-0.015,0.031) | | 0.0 |  |
|  | >500 | 6 | 0.005(-0.001,0.011) | | 0.0 | 6 | -0.008(-0.017,0.001) | | 0.0 |  |
| **Region** | Asia | 2 | 0.004(-0.021,0.028) | | 0.0 | 2 | -0.004(-0.003,0.022) | | 0.0 |  |
|  | North America | 4 | 0.004(-0.003,0.011) | | 0.0 | 5 | -0.009(-0.020,0.002) | | 0.0 |  |
|  | Europe | 3 | 0.004(-0.010,0.017) | | 0.0 | 3 | -0.001(-0.016,0.014) | | 0.0 |  |
| **Adjust for BMI** | yes | 2 | 0.004(-0.021,0.028) | | 0.0 | 3 | -0.002(-0.027,0.024) | | 0.0 |  |
|  | no | 7 | 0.004(-0.002,0.010) | | 0.0 | 7 | -0.006(-0.015,0.003) | | 0.0 |  |
| **Sex** | male | 5 | -0.001(-0.011,0.010) | | 13.3 | 6 | -0.000(-0.024,0.024) | | 41.2 |  |
|  | female | 5 | 0.008(-0.011,0.026) | | 50.6 | 6 | -0.006(-0.020,0.007) | | 0.0. |  |
| **Age** | <19 | 4 | 0.004(-0.010,0.018) | | 0.0 | 4 | -0.001(-0.016,0.014) | | 0.0 |  |
|  | ≥19 | 5 | 0.004(-0.003,0.011) | | 0.0 | 6 | -0.008(-0.018,0.002) | | 0.0 |  |

**Table S4.** Subgroup associations of PFDA exposure with thyroid health effects.

| Subgroup |  | | **TSH** | | | | | **T3** | | | | | | **T4** | | | | |
| --- | --- | --- | --- | --- | --- | --- | --- | --- | --- | --- | --- | --- | --- | --- | --- | --- | --- | --- |
|  |  | | **NO** | | **β(95%CI)** | | **I^2^** | **NO** | | **β(95%CI)** | | **I^2^** | | **NO** | **β(95%CI)** | | **I^2^** | |
| **Sample size** | <500 | | 4 | | -0.010(-0.078,0.059) | | 21.7 | 1 | | 0.020(-0.035,0.075) | | 0 | | 1 | 0.102(-0.228,0.432) | | 0 | |
|  | >500 | | 2 | | 0.002(-0.074,0.078) | | 52.9 | 1 | | -0.001(-0.022,0.019) | | -- | | 2 | -0.010(-0.026,0.005) | | 0 | |
| **Region** | Asia | | 2 | | 0.002(-0.074,0.078) | | 52.9 | 1 | | -0.001(-0.022,0.019) | | -- | | 2 | -0.010(-0.026,0.005) | | 0 | |
|  | North America | | 1 | | 0.210(-0.260,0.680) | | -- | 0 | | -- | | -- | | 0 | -- | | -- | |
|  | Europe | | 3 | | -0.018(-0.093,0.058) | | 35.3 | 1 | | 0.040(-0.040,0.120) | | -- | | 1 | -0.080(-0.660,0.500) | | -- | |
| **Adjust for BMI** | yes | | 3 | | 0.005(-0.066,0.077) | | 34.2 | 1 | | -0.001(-0.022,0.019) | | -- | | 2 | -0.010(-0.026,0.005) | | 0 | |
|  | no | | 2 | | -0.078(-0.186,0.029) | | 0.0 | 1 | | 0.040(-0.040,0.120) | | -- | | 1 | -0.080(-0.660,0.500) | | -- | |
| **Sex** | male | | 2 | | -0.029(-0.147,0.089) | | 66.5 | 1 | | 0.040(-0.040,0.120) | | -- | | 1 | -0.080(-0.660,0.500) | | -- | |
|  | female | | 1 | | -0.026(-0.205,0.153) | | -- | 0 | | -- | | -- | | 0 | -- | | -- | |
| **Age** | <19 | | 3 | | -0.018(-0.093,0.058) | | 35.3 | 1 | | 0.040(-0.040,0.120) | | -- | | 1 | -0.080(-0.660,0.500) | | -- | |
|  | ≥19 | | 2 | | **-0.022(-0.041,-0.003)** | | 0.0 | 1 | | -0.001(-0.022,0.019) | | -- | | 1 | -0.007(-0.027,0.130) | | -- | |
|  |  | |  | |  | |  |  | |  | |  | |  |  | |  | |
| subgroup | |  | | **FT3** | | | | | | | **FT4** | | | | | | |  |
|  | |  | | **NO** | | **β(95%CI)** | | | **I^2^** | | **NO** | | **β(95%CI)** | | | **I^2^** | |  |
| **Sample size** | | <500 | | 2 | | **-0.032(-0.056,-0.007)** | | | 0.0 | | 3 | | 0.014(-0.012,0.041) | | | 0.0 | |  |
|  |  | >500 | | 2 | | 0.023(-0.068,0.114) | | | 57.5 | | 2 | | 0.003(-0.016,0.023) | | | 0.0 | |  |
| **Region** | | Asia | | 2 | | 0.023(-0.068,0.114) | | | 57.5 | | 2 | | 0.003(-0.016,0.023) | | | 0.0 | |  |
|  |  | North America | | 0 | | -- | | | -- | | 1 | | 0.090(-0.020,0.210) | | | -- | |  |
|  |  | Europe | | 2 | | **-0.032(-0.056,-0.007)** | | | 0.0 | | 2 | | 0.010(-0.017,0.037) | | | 0.0 | |  |
| **Adjust for BMI** | | yes | | 2 | | 0.023(-0.068,0.114) | | | 57.5 | | 3 | | 0.011(-0.024,0.046) | | | 7.6 | |  |
|  | | no | | 2 | | **-0.032(-0.056,-0.007)** | | | 0.0 | | 2 | | 0.010(-0.017,0.037) | | | 0.0 | |  |
| **Sex** | | male | | 2 | | -0.007(-0.114,0.100) | | | 39.3 | | 2 | | 0.013(-0.024,0.049) | | | 0.0 | |  |
|  | | female | | 2 | | -0.006(-0.082,0.069) | | | 24.4 | | 2 | | 0.007(-0.034,0.048) | | | 0.0 | |  |
| **Age** | | <19 | | 3 | | 0.020(-0.06,0.021) | | | 46.5 | | 3 | | 0.010(-0.017,0.0370) | | | 0.0 | |  |
|  | | ≥19 | | 1 | | -0.007(-0.026,0.011) | | | -- | | 2 | | 0.027(-0.049,0.104) | | | 53.2 | |  |

**Table S5.** Subgroup associations of PFOS exposure with thyroid health effects.

| Subgroup |  | **TSH** | | | **T3** | | | **T4** | | |
| --- | --- | --- | --- | --- | --- | --- | --- | --- | --- | --- |
|  |  | **NO** | **β(95%CI)** | **I^2^** | **NO** | **β(95%CI)** | **I^2^** | **NO** | **β(95%CI)** | **I^2^** |
| **Sample size** | <500 | 7 | -0.001(-0.069,0.068) | 35.3 | 3 | 0.118(-1.745,1.981) | 19.8 | 3 | 0.171(-0.374,0.716) | 82.6 |
|  | >500 | 10 | -0.008(-0.016,0.001) | 0 | 5 | -0.008(-0.034,0.017) | 53.6 | 6 | -0.006(-0.018,0.006) | 0 |
| **Region** | Asia | 2 | -0.005(-0.063,0.052) | 29.4 | 2 | -0.017(-0.046,0.011) | 39.8 | 3 | -0.011(-0.027,0.005) | 30.7 |
|  | North America | 7 | 0.000(-0.042,0.045) | 0 | 5 | -0.016( -0.052,0.020) | 88.6 | 6 | -0.014(-0.063, 0.035) | 83.3 |
|  | Europe | 8 | -0.005(-0.015,0.005) | 0 | 1 | 0.03(-0.04,0.1) | -- | -- | -- | -- |
| **Adjust for BMI** | yes | 9 | -0.008(-0.016,0.001) | 0 | 2 | -0.027(-0.064,0.009) | 92 | 4 | -0.035(-0.110,0.04) | 89.5 |
|  | no | 8 | -0.006(-0.005,0.037) | 30.5 | 6 | 0.015(-0.05,0.08) | 33 | 5 | 0.083(-0.098,0.263) | 69.5 |
| **Sex** | male | 14 | -0.005(-0.012,0.002) | 0 | 4 | 0.012(-0.068,0.093) | 57.2 | 3 | 0.008(-0.032,0.048) | 0 |
|  | female | 12 | -0.001(-0.007,0.006) | 0.8 | 3 | -1.573(-6.233,3.088) | 83.3 | 3 | 0.011(-0.018,0.04) | 0 |
| **Age** | <19 | 7 | -0.006(-0.02,0.008) | 5.1 | 1 | 0.03(-0.04,0.1) | -- | 2 | 0.149(-0.203,0.501)51.7 | 51.7 |
|  | ≥19 | 8 | -0.008(-0.021,0.006) | 5 | 7 | -0.016(-0.052,0.02) | 84.1 | 7 | -0.009(-0.093,0.076) | 85.1 |

| subgroup |  | **FT3** | | | **FT4** | | |
| --- | --- | --- | --- | --- | --- | --- | --- |
|  |  | **NO** | **β(95%CI)** | **I^2^** | **NO** | **β(95%CI)** | **I^2^** |
| **Sample size** | <500 | 3 | -0.004(-0.038,0.03) | 53.7 | 5 | 0.015(-0.005,0.035) | 0 |
|  | >500 | 6 | 0.001(-0.006,0.008) | 29.4 | 6 | -0.006(-0.022,0.009) | 52.9 |
| **Region** | Asia | 2 | 0.06(-0.107,0.228) | 70.1 | 2 | 0.09(-0.211,0.391) | 47.8 |
|  | North America | 4 | -0.001(-0.008,0.007) | 16.2 | 6 | 0.003(-0.015,0.021) | 26.7 |
|  | Europe | 3 | -0.003(-0.027,0.02) | 61.2 | 3 | -0.004(-0.033,0.024) | 69.2 |
| **Adjust for BMI** | yes | 3 | 0.008(-0.015,0.032) | 60 | 4 | -0.011(-0.039,0.018) | 60 |
|  | no | 6 | -0.002(-0.009,0.005) | 11.4 | 7 | 0.005(-0.009,0.019) | 20 |
| **Sex** | male | 9 | -0.003(-0.007,0.002) | 42.2 | 10 | 0.001(-0.004,0.006) | 28.6 |
|  | female | 9 | -0.000(-0.003,0.003) | 25.2 | 10 | 0.000(-0.001,0.002) | 0 |
| **Age** | <19 | 4 | -0.001(-0.029,0.027) | 63.8 | 4 | -0.002(-0.033,0.028) | 65 |
|  | ≥19 | 5 | -0.001(-0.007,0.005) | 0 | 7 | 0.003(-0.01,0.016) | 12.3 |

**Table S6.** Subgroup associations of PFHxS exposure with thyroid health effects.

| Subgroup | |  | | | **TSH** | | | | | | **T3** | | | | | | | **T4** | | | | | |
| --- | --- | --- | --- | --- | --- | --- | --- | --- | --- | --- | --- | --- | --- | --- | --- | --- | --- | --- | --- | --- | --- | --- | --- |
|  | |  | | | **NO** | | **β(95%CI)** | | **I^2^** | | **NO** | | **β(95%CI)** | | **I^2^** | | | **NO** | **β(95%CI)** | | **I^2^** | | |
| **Sample size** | | <500 | | | 7 | | 0.027(-0.015,0.069) | | 21.6 | | 1 | | 0.030(-0.010,0.070) | | -- | | | 2 | 0.025(-0.136,0.187) | | 0.0 | | |
|  |  | >500 | | | 9 | | -0.001(-0.014,0.011) | | 0.0 | | 4 | | 0.008(-0.068,0.083) | | 88.7 | | | 5 | 0.012(-0.016,0.040) | | 68.6 | | |
| **Region** | | Asia | | | 2 | | -0.004(-0.023,0.014) | | 0.0 | | 1 | | -0.020(-0.032,0.000) | | -- | | | 2 | 0.002(-0.014,0.019) | | 23.9 | | |
|  |  | North America | | | 5 | | 0.004(-0.051,0.060) | | 0.0 | | 3 | | 1.207(-0.961,3.375) | | 89.2 | | | 4 | 0.050(-0.063,0.163) | | 71.5 | | |
|  |  | Europe | | | 9 | | 0.006(-0.018,0.030) | | 45.3 | | 1 | | 0.030(-0.010,0.070) | | -- | | | 1 | 0.103(-0.140,0.410) | | -- | | |
| **Adjust for BMI** | | yes | | | 10 | | 0.003(-0.013,0.019) | | 16.9 | | 1 | | -0.020(-0.032,0.000) | | -- | | | 3 | 0.003(-0.012,0.017) | | 0.0 | | |
|  | | no | | | 6 | | 0.004(-0.032,0.041) | | 15.6 | | 4 | | 0.037(-0.060,0.134) | | 83.9 | | | 4 | 0.076(-0.046,0.199) | | 72.6 | | |
| **Sex** | | male | | | 13 | | -0.000(-0.010,0.009) | | 12.3 | | 3 | | 0.020(-0.002,0.042) | | 0 | | | 3 | -0.002(-0.032,0.029) | | 0.0 | | |
|  | | female | | | 12 | | 0.003(-0.008,0.015) | | 8.1 | | 2 | | 1.944(-2.012,5.899) | | 94.6 | | | 2 | 0.135(-0.086,0.355) | | 87.4 | | |
| **Age** | | <19 | | | 6 | | 0.008(-0.022,0.038) | | 39.4 | | 1 | | 0.030(-0.010,0.070) | | -- | | | 2 | 0.025(-0.136,0.187) | | 0.0 | | |
|  | | ≥19 | | | 4 | | -0.005(-0.023,0.013) | | 0.0 | | 2 | | 1.919(-2.087,5.926) | | 94.7 | | | 3 | 0.003(-0.012,0.017) | | 0.0 | | |
|  | | |  | | |  | | | | | | | |  | | | | | | | |  |  |
| subgroup | | |  | | | **FT3** | | | | | | | | **FT4** | | | | | | | |  |  |
|  | | |  | | | **NO** | | **β(95%CI)** | | | | **I^2^** | | **NO** | | **β(95%CI)** | | | | **I^2^** | |  |  |
| **Sample size** | | | <500 | | | 4 | | 0.006(-0.015,0.027) | | | | 30.5 | | 4 | | 0.001(-0.018,0.019) | | | | 0.0 | |  |  |
|  |  |  | >500 | | | 5 | | 0.005(-0.005,0.015) | | | | 47.4 | | 5 | | -0.008(-0.017,0.001) | | | | 0.0 | |  |  |
| **Region** | | | Asia | | | 2 | | 0.143(-0.188,0.475) | | | | 84.8 | | 2 | | 0.005(-0.074,0.087) | | | | 2.4 | |  |  |
|  |  |  | North America | | | 3 | | 0.006(-0.001,0.130) | | | | 0.0 | | 4 | | -0.010(-0.020,0.000) | | | | 0.0 | |  |  |
|  |  |  | Europe | | | 4 | | 0.006(-0.015,0.027) | | | | 30.5 | | 3 | | -0.001(-0.022,0.020) | | | | 17.8 | |  |  |
| **Adjust for BMI** | | | yes | | | 4 | | 0.015(-0.015,0.044) | | | | 67.0 | | 5 | | -0.001(-0.014,0.030) | | | | 0.0 | |  |  |
|  | | | no | | | 5 | | 0.005(-0.001,0.011) | | | | 0.0 | | 3 | | -0.011(-0.021,-0.001) | | | | 0.0 | |  |  |
| **Sex** | | | male | | | 9 | | 0.000(-0.002,0.003) | | | | 10.1 | | 9 | | -0.001(-0.006,0.003) | | | | 58.2 | |  |  |
|  | | | female | | | 9 | | 0.001(-0.002,0.004) | | | | 33.6 | | 10 | | 0.001(-0.001,0.003) | | | | 0.0 | |  |  |
| **Age** | | | <19 | | | 3 | | 0.009(-0.064,0.083) | | | | 71.7 | | 2 | | 0.021(-0.013,0.055) | | | | 0.0 | |  |  |
|  | | | ≥19 | | | 4 | | -0.001(-0.007,0.005) | | | | 0.0 | | 4 | | -0.007(-0.019,0.005) | | | | 16.0 | |  |  |
|  | |  | | |  | | | | | |  | | | | | | |  | | | | | |
